# Association of *KCNB1 *to rheumatoid arthritis via interaction with *HLA-DRB1*

**DOI:** 10.1186/1753-6561-3-s7-s134

**Published:** 2009-12-15

**Authors:** Xiangjun Xiao, Yufang Zhang, Kai Wang

**Affiliations:** 1 Program of Public Health Genetics, University of Iowa, 200 Hawkins Drive E177 GH, Iowa City, Iowa 52242, USA; 2 Department of Biostatistics, University of Iowa, 200 Hawkins Drive C227 GH, Iowa City, Iowa 52242, USA

## Abstract

With the rapid development of large-scale high-throughput genotyping technology, genome-wide association studies have become a popular approach to mapping genes underlying common human disorders. Some genes are discovered, but many more have not been. Because these genes were not initially identified, it is reasonable to assume that their main effect is weak. We propose a method to accommodate such a situation. It is applied to the Genetic Analysis Workshop 16 Problem 1 case-control data in which shared-epitope alleles of *HLA-DRB1 *show very strong association with rheumatoid arthritis. Because some previous functional studies have reported association of gene *KCNB1 *to rheumatoid arthritis, we evaluate whether the gene *KCNB1 *contributes to the genetics of rheumatoid arthritis in this data set. Fifteen single-nucleotide polymorphisms from this gene were chosen. The association of *KCNB1 *gene to rheumatoid arthritis seems to be moderate.

## Background

Conventional methods such as linear regression and logistic regression are widely used in genetic association tests in the presence of gene-gene or gene-environment interaction. In recent years several new methods have been developed. Based on Tukey's non-additivity model, Chatterjee et al. [1] proposed a model that makes use of "generalized association parameters" to map additional genes while allowing for gene-gene and gene-environment interactions. This method may have higher power to detect genetic variants than some existing methods. One drawback of their work is that it does not accommodate the situation in which the main effect of the second gene is bounded by constant times its interaction effect with the first gene. To overcome this drawback, Wang [2] proposed a method and derived the asymptotic distribution of the likelihood-ratio test statistic. This method allows the main effect of the second gene to be bounded but does not assume it to be zero. However, the method proposed by Wang is for a continuous phenotype. In this report, we generalized that method to case-control study so it can be applied to the Genetic Analysis Workshop 16 (GAW16) Problem 1 data.

We choose to study genes *HLA-DRB1 *using its shared-epitope alleles and *KCNB1*. The relationship between *HLA-DRB1 *and increased risk for rheumatoid arthritis has been studied for 20 years, and several high risk alleles of *HLA-DRB1 *are treated as shared-epitope alleles [3,4], which are used widely in association study for rheumatoid arthritis [5]. There are also some studies covering regions including genes *PTPN22 *and *TRAF-C5*, and at least two alleles of *PTPN22 *were associated with increased risk for rheumatoid arthritis [6]. In a genome-wide study, *TRAF-C5 *was found to be a risk locus for rheumatoid arthritis [7]. Unlike *PTPN22 *and *TRAF-C5*, which are supported by statistical evidence, there is some functional evidence supporting the active role of *KCNB1 *in rheumatoid arthritis. The functional channel in human T lymphocytes is composed of four identical *KCNB1 *sub-units, and several peptide inhibitor of *KCNB1 *have been developed as therapy for autoimmune diseases [8] such as type 1 diabetes mellitus and rheumatoid arthritis. An expression study [9] found that there is a downregulation of potassium channels including *KCNB1 *in autoimmune diseases.

Because the association signal of *KCNB1 *is not very strong (it has not been discovered by other genome-wide association studies), we conjecture that its effect may mainly be manifested through its interaction with the *HLA-DRB1 *gene. The goal of the current analysis is to investigate whether the gene *KCNB1*, a previously reported gene associated with rheumatoid arthritis, is associated with rheumatoid arthritis in this GAW16 dataset. For this purpose, we use two statistical techniques, including one particularly developed for this study, which may be helpful in mapping genes that have weak main effect.

## Methods

The GAW16 Problem 1 data comes from the North American Rheumatoid Arthritis Consortium (NARAC) and includes 868 cases and 1194 controls. Single-nucleotide polymorphisms (SNPs) of all individuals were genotyped using 550 k Illumina chip (*n *= 545,080). A genome-wide association scan based on the trend test was performed to assess the association of all SNPs to rheumatoid arthritis.

The regular logistic regression of two-locus disease model with interaction is logit(*π *(*x*)|*SE*, *SNP*) = *α*_0 _+ *α*_1 _× *SE *+ *α*_2 _× *SNP *+ *α*_3 _× *SE *× *SNP*, where SE is the number of shared-epitope alleles of *HLA-DRB1 *and SNP is the number of a chosen allele at an SNP of *KCNB1*, and the coefficients *α*_2 _and *α*_3 _measure the additional contribution of locus 2 over that of locus 1. A traditional test of both effects with two degrees of freedom is used to assess the association of the second locus, and the hypotheses to be tested are *H*_0_: *α*_2 _= *α*_3 _= 0 vs. *H*_1_: *α*_2 _≠ 0 or *α*_3 _≠ 0. This test treats the coefficients *α*_2 _and *α*_3 _as unrelated to each other because one of two coefficients can be 0 regardless of the values of the other one. However, this test may not be effective because the values of *α*_2 _and *α*_3 _could depend on whether there is association due to locus 2.

The situation of interest in this study is that main effect |*α*_2_|, is constrained by the interaction |*α*_3_| effect. The constraint is *α*_3 _≠ 0, |*α*_2_| ≤ *M *× |*α*_3_|, with *M *a pre-specified constant, and then the hypotheses to test in this study are *H*_0_: *α*_2 _= *α*_3 _= 0 vs. *H*_1 _: *α*_3 _≠ 0, |*α*_2_| ≤ *M *× |*α*_3_|. Based on this constraint, two extreme situations need to be considered. When *M *is set to be 0, *α*_2 _has to be 0 as well, and then the test measures interaction effect only (H_1 _: *α*_2 _= 0, *α*_3 _≠ 0) with 1 df; when *M *is very large, *α*_2 _is not affected by *α*_3_, and then the test measures both effects (*H*_1 _: *α*_2 _≠ 0 or *α*_3 _≠ 0) with 2 df.

An appropriate value for *M *should achieve a balance between measuring interaction only and both effects. Based on some simulation results in linear regression [2], when *M *is in the range of [0.1, 0.3], the asymptotic quantiles of statistic based on this test seem to be rather different from those of the interaction-only test and the both-effects test. We set *M *to 0.4 in this analysis because the value of *M *should reflect the moderate association of *KCNB1 *and it should not be too small. Because the distribution of the likelihood-ratio statistic of this proposed test is unknown for case-control study, and in order to compare *p*-values, the estimation of the *p*-values need to be based on the same procedure. Ten thousand permutations are used to estimate the *p*-values in three tests for each SNP.

We also use principal-component analysis to test multiple SNPs in *KCNB1 *jointly. The principal components were obtained via the statistical package R (version 2.6.2) based on the correlation coefficient matrix.

## Results

The result of a genome-wide association scan is consistent with previous studies [8], with the strongest signal coming from HLA region on 6p21. For genes *HLA-DRB1 *and *KCNB1*, a *p*-value < 10^-100 ^for *HLA-DRB1 *and the range of *p*-values for *KCNB1 *from 10^-4 ^to 10^-7 ^were set as thresholds for significance.

*KCNB1 *(potassium voltage-gated channel, Shab-related subfamily; member 1) is 110,667 bp long; mRNA is 3756 bp long and encodes a protein of 858 amino acids. Position information comes from *Homo sapiens *chromosome 20 genomic contig, NT_011362.9. There are 36 SNPs available in *KCNB1 *for this dataset, rs1051295 (A/G) is located in 3'UTR and all others are located in introns. The most significant 15 SNPs from this gene in the initial scan were selected in subsequent analysis. The first principal component accounts for about 80% of variation for 15 SNPs. The proposed method was applied to these 15 SNPs and the first component (Table 1). Also presented in Table 1 are two tests: interaction effect only test (*H*_1 _: *α*_2 _= 0, *α*_3 _≠ 0), and both effects test (*H*_1 _: *α*_2 _≠ 0 or *α*_3 _≠ 0). In Table 1, *p*-values show that the interaction-effect-only test and the proposed test are more significant than the both-effects test, and the overall strengths of these two tests are similar. *p*-Values of six SNPs based on the proposed test are smaller than those of interaction-only test; *p*-values of four SNPs based on interaction-only test are smaller than those of the proposed test; *p*-values of two SNPs are the same. Because of limitation of the number of permutations, *p*-values of three SNPs and the first principal component cannot be compared (*p*-value < 0.0001).

**Table 1:** *p*-Values in three association tests based on 10,000 permutations

SNP	ID	Position	function	Interaction effect	Both effects	Proposed model
1	rsl051295	47,422,312	3'UTR	0.0008	0.0018	0.0008
2	rs2426154	47,435,402	intron	0.0011	0.0017	0.0008
3	rs237485	47,437,645	intron	0.0008	0.0015	0.0008
4	rs1961192	47,439,714	intron	0.0004	0.0005	0.0003
5	rs9636516	47,440,607	intron	0.0007	0.0017	0.0012
6	rs6063397	47,449,616	intron	0.0011	0.0023	0.0008
7	rs2057077	47,457,043	intron	0.0011	0.0017	0.0005
8	rs6067085	47,458,509	intron	0.0001	0.0011	0.0004
9	rs6090975	47,459,969	intron	<0.0001	<0.0001	<0.0001
10	rs6125647	47,460,870	intron	0.0003	0.0004	<0.0001
11	rs926673	47,463,417	intron	<0.0001	<0.0001	<0.0001
12	rs237462	47,466,610	intron	0.0019	0.0035	0.0021
13	rs237476	47,484,789	intron	0.0052	0.0102	0.0076
14	rs742758	47,495,219	intron	<0.0001	<0.0001	<0.0001
15	rs572845	47,510,021	intron	0.0027	0.0022	0.0007
First principal component				<0.0001	<0.0001	<0.0001

## Discussion

The association between *HLA-DRB1 *gene and rheumatoid arthritis is very strong. Except for some known genes, a genome-wide scan found no other genes that show obvious association. One possibility is that other genes have a weak main effect, making them hard to detect. We propose a method for case-control study that allows the main effect of the second gene to be weak relative to its interaction effect with the first gene. Using this model, we studied the association between *KCNB1 *and rheumatoid arthritis.

The results of three tests used in this study support our assumption that the effect of *KCNB1 *may mainly be manifested through its interaction with the *HLA-DRB1 *gene.

The interaction-effect only test (*M *= 0) and the proposed test (*M *= 0.4) perform better than the both-effects test; for six SNPs, the proposed test even performs better than interaction-effect-only test.

The *M *value reflects the strength of main effect in the proposed model. *M *values of 0.1 and 0.4 represent small effect and moderate effect, respectively. Considering the moderate main effect of *KCNB1*, we set *M *value to be 0.4. The *M *value should be determined by researchers, every gene has different *M *value. A wrongly chosen *M *value will reduce the power in the test, so we suggest that a rough range of *M *values should be determined by some prior information, and then a number of interations be used to get the appropriate *M *value. Computing burden is a big concern in case-control study. When the number of permutations is larger than 10,000, comparisons cannot be made, so a more efficient algorithm for permutation needs to be developed. This method is more applicable in linear regression because the asymptotic distribution of the likelihood-ratio test statistic has been derived [2].

Previous studies have reported association of rheumatoid arthritis to *KCNB1 *gene. Based on our analysis, the association strength between *KCNB1 *and rheumatoid arthritis seems to be moderate in the GAW16 Problem 1 data.

## Conclusion

We used two methods, including one developed by us, to investigate the association between *KCNB1 *gene and rheumatoid arthritis. Based on results, the strength of the association is moderate. This association needs further confirmation.

## List of abbreviations used

GAW16: Genetic Analysis Workshop 16; SNP: Single-nucleotide polymorphism.

## Competing interests

The authors declare that they have no competing interests.

## Authors' contributions

XX participated in the design of the study and performed the statistical analysis. KW conceived of the study, participated in its design and coordination, and helped to draft the manuscript. YZ participated in the design of the study. All authors read and approved the final manuscript.
